# Emergence of *Trichosporon mycotoxinivorans (Apiotrichum mycotoxinivorans)* invasive infections in Latin America

**DOI:** 10.1590/0074-02760170011

**Published:** 2017-10

**Authors:** João Nobrega de Almeida, Elaine Cristina Francisco, Maria Goreth M de Andrade Barberino, Luiz Vicente Ribeiro F da Silva, Oriana M Brandão, Arnaldo Lopes Colombo, Ana Carolina Barbosa Padovan

**Affiliations:** 1 Universidade de São Paulo, Instituto de Medicina Tropical, Divisão de Laboratório Central (LIM03) e Laboratório de Micologia Médica (LIM53), Hospital das Clínicas da Faculdade de Medicina da Universidade de São Paulo, São Paulo, SP, Brasil; Universidade de São Paulo, Hospital das Clínicas, Faculdade de Medicina, Universidade de São Paulo, São Paulo, SP, Brasil; 2 Universidade Federal de São Paulo, Escola Paulista de Medicina, Laboratório Especial de Micologia, São Paulo, SP, Brasil; 3 Universidade Federal da Bahia, Complexo Hospitalar Universitário Professor Edgar Santos, Laboratório de Microbiologia, Salvador, BA, Brasil; 4 Universidade de São Paulo, Instituto da Criança, Hospital das Clínicas da Faculdade de Medicina da Universidade de São Paulo, São Paulo, SP, Brasil; 5 Universidade Federal da Bahia, Departamento de Medicina, Complexo Hospitalar Universitário Professor Edgar Santos, Salvador, BA, Brasil; 6 Universidade Federal de Alfenas, Departamento de Microbiologia e Imunologia, Alfenas, MG, Brasil

**Keywords:** Trichosporon mycotoxinivorans, Apiotrichum mycotoxinivorans, infection, biofilm, antifungal susceptibility, literature review

## Abstract

We report the first two cases of *Trichosporon mycotoxinivorans* infections in Latin America. We also conducted a literature review and a microbiological investigation, including that of clinical and environmental isolates. A 30-year-old man with chronic renal failure had disseminated infection after dialysis and a 15-year-old boy with cystic fibrosis (CF) had pulmonary exacerbations with positive respiratory samples. A review of the relevant literature revealed that deep-seated infections were related to immunosuppression or invasive devices, while most of the CF patients showed a decline in lung function after positive cultures. Phylogenetic analyses revealed three distinct circulating genotypes. MALDI-TOF mass spectrometry analysis showed similar spectral profiles and correctly identified all strains/isolates. Biofilm production was documented in a bloodstream isolate and biofilm-producing cells showed high minimum inhibitory concentrations against antifungals.

The basidiomycetous yeast, *Trichosporon mycotoxinivorans* (*T. mycotoxinivorans)* was first described in 2004 (Molnar et al. 2004). *T. mycotoxinivorans* has been proposed as a microbial feed additive against mycotoxins produced by moulds, protecting livestock from intoxication after consumption of contaminated crop (Politis et al. 2005). Moreover, in Brazil, *T. mycotoxinivorans* was recovered from effluents of the dairy industry (Monteiro et al. 2010) and has been studied for the bioconversion of biodiesel refinery waste (Monteiro et al. 2012). However, since 2009, this microorganism has been considered a new pathogen for patients with cystic fibrosis (CF) (Hickey et al. 2009). In 2015, Liu and collaborators conducted an in-depth phylogenetic reclassification of the tremellomycetous yeasts, proposing a new genus name to *T. mycotoxinivorans*, and that is *Apiotrichum mycotoxinivorans* (*A. mycotoxinivoran*s) (Liu et al. 2015).

In order to provide additional data to the current knowledge regarding this emergent microorganism, we report clinical data regarding the first two cases of *T. mycotoxinivorans* (*A. mycotoxinivoran*s) deep-seated infections documented in Latin America, along with a brief literature review. Clinical isolates were characterised by IGS rDNA sequencing, proteomic profile (MALDI-TOF), and capability to produce biofilms.

In December 2010, a 30-year-old male patient with a history of chronic renal failure was admitted to a tertiary hospital in Salvador, presenting with altered level of consciousness after a peritoneal dialysis session. On day 3, the patient was diagnosed with sepsis, and blood and peritoneal fluid cultures were collected for microbiological investigation. The peritoneal fluid became positive for *Trichosporon* sp. after two days of incubation (day 5). Despite antifungal therapy [amphotericin B (AMB) deoxycholate, 40 mg/day] prescribed on day 5, the patient’s fever persisted, and he developed multiple pulmonary infiltrates and respiratory failure. Blood cultures collected on day 12 also became positive for *Trichosporon* spp. (isolate L7537) and antifungal therapy was changed to voriconazole on day 14 (300 mg q 12 h on the first day, then 200 mg q 12 h). The attempts to remove the Tenckhoff and haemodialysis catheters were unsuccessful, and the patient’s condition deteriorated due to sepsis by *Chryseobacterium meningosepticum*, and he died on day 29.

The second case was a 15-year-old boy from a city near São Paulo (Guaratinguetá) with a diagnosis of CF (CFTR genotype F508del and G542X) and multiple pulmonary exacerbations, receiving intermittent treatment with antibiotics. In September 2014, another pulmonary exacerbation was diagnosed with a significant decline in lung function (42 to 28% of predicted forced expiratory volume in the first second - FEV1). At that time (day 0), the sputum culture became positive for *Trichosporon* spp. (isolate L998/16). Due to the persistent positive sputum cultures and since the FEV1 values remained below 40%, a long course therapy with fluconazole (150 mg/day for 21 days) was started (day 210). Sputum cultures became negative (days 300, 390, 480) and the patient exhibited partial recovery of the lung function (FEV1 39%). However, sputum cultures became positive for *Trichosporon* spp. once again (day 600) and antifungal treatment is being envisaged in case of a new episode of pulmonary exacerbation.

Both clinical isolates (L7537 and L998/16) were identified as *T. mycotoxinivorans (A. mycotoxinivorans)* after sequence analysis of the IGS1 region from the rDNA (see microbiological investigation below). In accordance with the standard definitions of opportunistic invasive fungal infections (de Pauw et al. 2008) adapted by Colombo et al. (2011) for investigating *Trichosporon* spp*.* infections, we considered case one and case two as proven and probable deep-seated trichosporonosis, respectively.

As detailed in the supplementary material (Supplementary data, Table), only four cases of *T. mycotoxinivorans* fungaemia have been reported thus far, one CF patient after lung transplantation, one patient with acute promyelocytic leukaemia and neutropaenia, and another two critically ill patients who were exposed to invasive medical procedures (Hirschi et al. 2012, Dabas et al. 2016). All these patients died despite administration of antifungal therapy, which consisted of initial liposomal amphotericin B (AMB) replaced by voriconazole (n = 1), voriconazole monotherapy (n = 1), caspofungin monotherapy (n = 1), and deoxycholate AMB monotherapy (n = 1).

The distinction of pulmonary colonisation or infection by *T. mycotoxinivorans* (*A. mycotoxinivorans*) in non-immunosuppressed CF patients is a matter of debate that has been recently raised by different investigators. According to Esther Jr et al. (2016), CF patients with respiratory samples positive for *T. mycotoxinivorans* may exhibit lung function decline over time when compared to CF patients with negative cultures. These findings suggest that this microorganism may not be simply a coloniser of the respiratory tract of CF patients. Indeed, among eight well-documented cases of non-immunosuppressed CF patients reported with positive respiratory samples for *T. mycotoxinivorans* (*A. mycotoxinivorans*) (Supplementary data, Table), six showed a decline in lung function after the first positive culture (Kröner et al. 2013, Shah et al. 2014, Goldenberger et al. 2016, Muñiz et al. 2016). Consolidating our case with the information provided by the literature review summarised above, we suggest that *T. mycotoxinivorans* (*A. mycotoxinivorans*) may colonise and infect the respiratory tract of CF patients, causing progressive deterioration of their lung function.

Finally, we carried out a microbiological analysis of clinical (L7537, L998/16) and environmental (L1024/16)isolates, and reference strains (CBS9756 and CBS10094) of *T. mycotoxinivorans* (*A. mycotoxinivorans*). The microbiological analysis included investigation of genetic and proteomic diversity, as well as of biofilm production as a predictor of virulence (Rajendran et al. 2016) and antifungal resistance. Sequence analysis of the IGS1 region from the rDNA of the isolates and strains were carried out using a standard protocol (Sugita et al. 2002), and a phylogenetic tree was constructed using MEGA software version 7.0, with the addition of IGS1 rDNA sequences of *Apiotrichum* spp. and *Trichosporon* spp. retrieved from the GenBank database. Protein profiles were generated by MALDI-TOF mass spectrometry (MicroFlex, Bruker Daltonics, Bremen, Germany) according to the manufacturer’s recommendations [ethanol (70%) and formic acid (70%) extraction protocol]. Data analysis was performed using the Biotyper™ 3.1 software (Bruker Daltonics), with log score ≥ 2 considered as correct for species identification. Biofilm production was quantified according to a previously described method (Iturrieta-González et al. 2014), using three *Trichosporon asahii* strains isolated from blood cultures as controls that were previously classified as low (L773), medium (L7730), and high biofilm producers (L2589). Briefly, biofilm formation was induced in 96-well polystyrene plates over 48 h, using RPMI-1640 medium. Quantification of mature biofilms was performed using the crystal violet staining method (Iturrieta-González et al. 2014). Two replicate plates on different occasions were assayed, totalling 16 wells analysed. Antifungal susceptibility tests (AST) were performed for amphotericin B (AMB), anidulafungin (ANI), fluconazole (FCZ), and voriconazole (VCZ). Against planktonic cells, AST was carried out with the reference broth microdilution method (CLSI 2008). For the biofilm-producing cells, AST was carried out according to the protocol described by Iturrieta-González et al. (2014).

The phylogenetic analysis of IGS1 sequences (Supplementary data, Fig. 1) confirmed the species identification by clustering our isolates in the *Apiotrichum* genus clade (bootstrap = 100). Furthermore, the *Apiotrichum* genus clade formed three distinct phylogenetic groups or genotypes: the first group obtained from the bloodstream isolated L7537 and the strain CBS10094; the Indian bloodstream isolates formed a second group which was closely related to the first one; a third group comprised of the isolate L998/16 recovered from the patient with CF and the type strain CBS9756, isolated from an insect and analysed as a microbial additive against mycotoxins in the food industry (Molnar et al. 2004). Despite its intra-specific genetic diversity, analysis by MALDI-TOF mass spectrometry showed that all isolates and strains of *T. mycotoxinivorans* (*A. mycotoxinivorans*) had similar spectral profiles and had correct species assignment with log score values > 2. A dendrogram based on the main spectrum profiles (MSP) from the Bruker’s database and from our isolates/strains gathered all *T. mycotoxinivorans* (*A. mycotoxinivorans*) MSPs in the same node (Supplementary data, Fig. 2). Four of five isolates/strains were characterised as low biofilm producers, in spite of some intraspecific variations. Strikingly, the bloodstream isolate was characterised as a medium biofilm producer (Figure). Susceptibility tests of planktonic cells at 48 h readings showed high MIC values for AMB and ANI for all isolates, with geometric means (GM) of 13.9 and 16 µg/mL, respectively, whereas FCZ and VCZ exhibited the best *in vitro* antifungal activities, with GM at 2.3 µg/mL and 0.12 µg/mL, respectively (Figure). Although azoles derivatives showed good *in vitro* activity against planktonic cells of *T. mycotoxinivorans* (*A. mycotoxinivorans*), our investigation suggests that microbial biofilms decreased their susceptibility to triazoles, which may partially influence the poor clinical response to antimicrobial therapy in *T. mycotoxinivorans* (*A. mycotoxinivorans*) infections.

**Figure:**
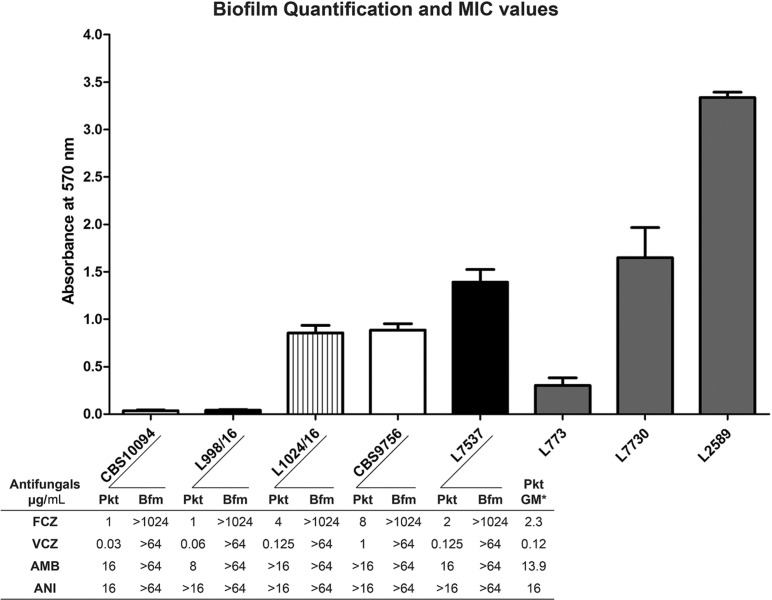
Biofilm quantification of *Trichosporon mycotoxinivorans* (*Apiotrichum mycotoxinivorans*) isolates and MIC values of planktonic (Pkt) and biofilm (Bfm) forming cells at 48 h readings. Reference strains are presented in white [CBS10094 and CBS9756 (isolate HB1175 from Molnar et al. 2004)], clinical isolates are in black (case 1: L7537 and case 2: L998/16), the environmental isolate is in the striped box (L1024/16) and *Trichosporon asahii* biofilm-control strains are in grey (L773, L7730 and L2589). The *T. asahii* biofilm quantification levels represent the threshold for detecting low, medium and high biofilm-forming isolates, respectively. Histograms depict the mean and standard deviation of 16 biofilm measurement replicates. *GM: Geometric Mean of MIC values from planktonic cells.

In conclusion, while still considered rare, *T. mycotoxinivorans* (*A. mycotoxinivorans*) may cause fungaemia and lung infections in patients exhibiting risk conditions for acquiring such infections. They are able to produce biofilms, with apparently negative impact on their susceptibility to triazoles. Finally, we provided new data about the genetic diversity of *T. mycotoxinivorans* (*A. mycotoxinivorans*), warranting further molecular epidemiological studies to better understand the distribution of the different molecular types of *T. mycotoxinivorans* (*A. mycotoxinivorans*).
