# Supplementary material for: Emergence of Trichosporon mycotoxinivorans (Apiotrichum mycotoxinivorans) invasive infections in Latin America
Source: Mem Inst Oswaldo Cruz. 2017 Oct;112(10):719–22. doi: 10.1590/0074-02760170011 (PMC5607521; doi:10.1590/0074-02760170011)
Supplement: Supplementary file 1 [file 0074-0276-mioc-112-10-0719-suppl01.pdf]

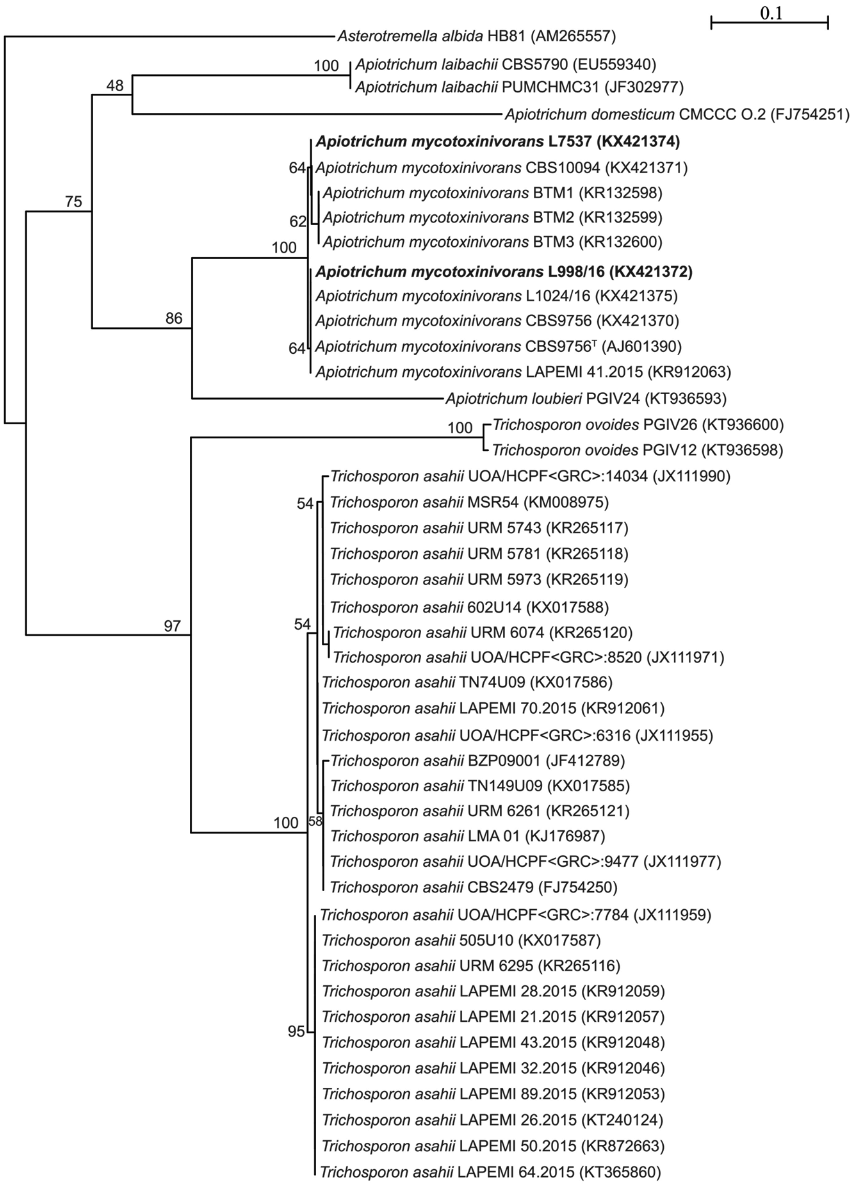

Fig. 1: phylogenetic analysis of the IGS1 rDNA sequences of *Trichosporon mycotoxinivorans* (*Apiotrichum mycotoxinivorans*) isolates and related species. The analysis was generated by Neighbour-Joining method using Kimura-2-Parameters as a model of nucleotides substitution. 1,000 bootstrap pseudoreplicate values are depicted on the main nodes. Species names are followed by strain numbers and GenBank accession numbers in parentheses. Sequences from clinical isolates characterised in this report are depicted in bold. T = type strain. Scale bar represents the number of substitutions per site.

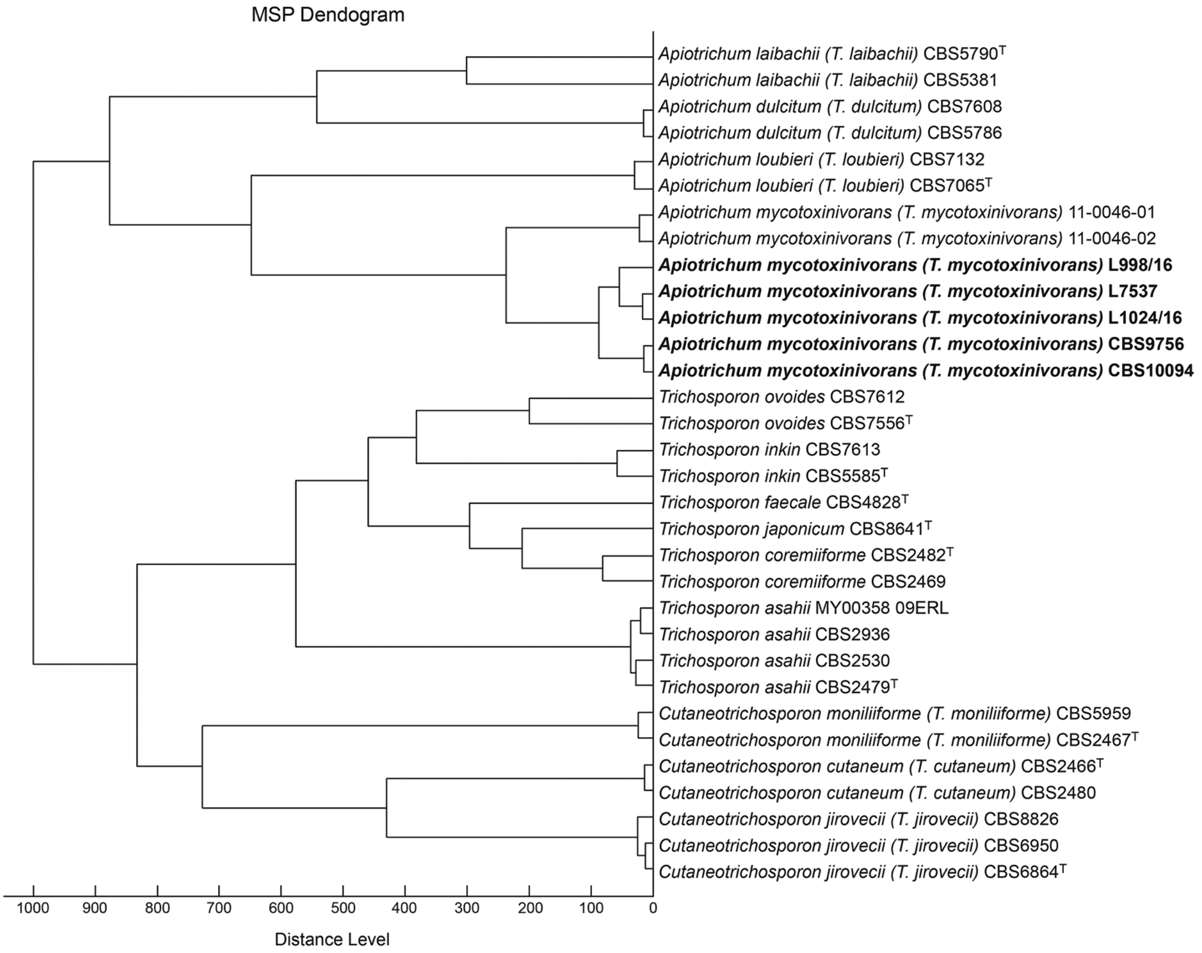

Fig. 2: Bruker Biotyper™ dendrogram clustering of the *Apiotrichum*, *Trichosporon* and *Cutaneotrichosporon* species main spectrum profiles (MSPs). The MSPs from the isolates and strains of this study are highlighted in bold. Distances were displayed in relative units on the *x* axis.

TABLE I

Clinical and microbiological characteristics of patients with positive cultures for *Apiotrichum mycotoxinivorans* reported in the literature and our cases

References	Age, gender, baseline disease	Site of isolation	Clinical presentation	Previous colonisation of the respiratory tract	Prior antimicrobial therapy	Antifungal susceptibility patern [MIC (mg/L)]	Treatment and outcome
Hickey et al. (2009)	20, male, cystic fibrosis, systemic corticosteroids	Respiratory tract	Pulmonary infection	MRSA, *Candida albicans*, BC	Piperacillin-tazobactam, cotrimoxazole	AMB 1-4, Flu 2-> 64, Vor 0.06-> 8	L-AMB, Vor, died
Hirschi et al. (2012)	35, male, cystic fibrosis, lung and liver transplant recipient	Blood, thoracic wound, heart, pericardium, and bronchi	Disseminated infection, co-infection with *Aspergillus fumigatus* and *Scedosporium apiospermum*	PA, *Aspergillus fumigatus*	Cas	NA	Vor, died
Kröner et al. (2013)	21, female, cystic fibrosis	Respiratory tract	Pulmonary exacerbation, decline of %FEV1	SM, PA, *Candida albicans, Escherichia coli, Aspergillus flavus*	Cephalexin, gentamicin	NA	Nebulised AMB, improvement of respiratory symptoms but without recovery of pulmonary function
Shah et al. (2014)	18, female, cystic fibrosis	Respiratory tract	Pulmonary exacerbation, decline of %FEV1	MSSA, PA, SM, *Serratia marscescens, Aspergillus fumigatus, Acinetobacter junii*	Azythromycin, inhaled tobramycin	NA	No, improvement of respiratory symptoms but without recovery of pulmonary function
Shah et al. (2014)	8, female, cystic fibrosis	Respiratory tract	Pulmonary exacerbation	MSSA, PA, *Flavobacterium breve*, SM	Azythromycin, inhaled tobramycin	NA	No, improvement of respiratory symptoms but without recovery of pulmonary function
Shah et al. (2014)	17, male, cystic fibrosis	Respiratory tract	Pulmonary exacerbation, decline %FEV1	MSSA, PA, SM, *Haemophilus influenzae*	Azythromycin, inhaled tobramycin	NA	Vor and nebulised AMB, improvement of respiratory symptoms but without recovery of pulmonary function
Shah et al. (2014)	17, male, cystic fibrosis	Respiratory tract	Pulmonary exacerbation, decline %FEV1	MSSA, PA, *Pseudomonas stutzeri, Pseudomonas putida, Pseudomonas fluorescens*	Azythromycin, inhaled tobramycin	NA	No, improvement of respiratory symptoms but without recovery of pulmonary function
Muñiz et al. (2016)	37, male, cystic fibrosis	Respiratory tract	Pulmonary colonisation	MSSA, PA	Itraconazole*	NA	No, resolved without antifungal treatment

References	Age, gender, baseline disease	Site of isolation	Clinical presentation	Previous colonisation of the respiratory tract	Prior antimicrobial therapy	Antifungal susceptibility patern [MIC (mg/L)]	Treatment and outcome
Goldenberger et al. (2016)	32, male, cystic fibrosis	Respiratory tract	Pulmonary exacerbation, decline %FEV1	MSSA, PA, SM	Azythromycin, trimethoprim/ sulfamethoxazol, piperacillin/ tazobactam, tobramycin inhaled amikacin	AMB 0.25-2, Flu 2-8, Vor 0.03-0.12, Itr 0.015-0.25	Vor, Itr, improvement of respiratory symptoms and stabilisation of pulmonary function
Dabas et al. (2016)	55, female, multiple myeloma, renal amyloidosis, chronic renal failure	Blood	Bloodstream infection	No	NA	NA	Cas, died
Dabas et al. (2016)	4, male, acute promyelocytic leukemia, neutropenia	Blood	Bloodstream infection	No	NA	NA	AMB, died
Dabas et al. (2016)	11, male, aortic valve homograft	Blood	Endocarditis, pulmonary embolism	No	NA	NA	L-AMB, Vor, died
Present report	30, male, chronic renal failure, pulmonary tuberculosis, peritoneal dialysis	Peritoneal fluid, blood	Peritonitis, bloodstream infection	No	Rifampicin, isoniazid, pyrazinamide, ethambutol, ceftazidime	AMB 16, Flu 2, Vor 0.125	AMB, Vor, died
Present report	15, male, cystic fibrosis	Respiratory tract	Pulmonary exacerbation, decline %FEV1	MSSA, PA	Inhaled tobramycin, ciprofloxacin, colistin, cotrimoxazole	AMB16, Flu 1, Vor 0.03	Flu, stabilisation of pulmonary function

AMB: amphotecin B; BC: *Burkholderia cepacia*; Cas: caspofungin; Flu: fluconazole; Itr: itraconazole; L-AMB: liposomal amphotericin; MIC: minimal inhibitory concentration; MRSA: methicillin-resistant *Staphylococcus aureus*; MSSA: methicillin-susceptible *Staphylococcus aureus*; NA: not available; PA: *Pseudomonas aeruginosa*; SM: *Stenotrophomonas maltophilia*; Vor: voriconazole; %FEV1: percentage of predicted forced expiratory volume in 1s; *: itraconazole prescribed after a positive respiratory sample for *Trichosporon mucoides*.
